# Risk appetite-enhanced game-theoretic approach for modelling mandatory lane-changing behaviour in freeway merging areas

**DOI:** 10.1371/journal.pone.0355386

**Published:** 2026-08-07

**Authors:** Bingtong Wang, Shunchao Wang, Gaili He

**Affiliations:** 1 School of Transportation, Southeast University, Nanjing, China; 2 Key Laboratory of the Coastal Zone Exploitation and Protection, Ministry of Natural Resources, Nanjing, China; 3 School of Automotive Engineering, Harbin Institute of Technology, Weihai, China; 4 School of Geography and Ocean Science, Nanjing University, Nanjing, China; Southwest Jiaotong University, CHINA

## Abstract

Addressing adverse effects on traffic safety resulting from mandatory lane changes presents a significant challenge, necessitating the development of an empirically accurate method that incorporates drivers’ risk appetite into modelling merging behaviour. Game theory serves as a commonly utilized approach for analysing interactive lane-changing behaviour and accurately predicting various vehicle actions. This study introduces a game-theoretical decision-making model tailored for mandatory lane changes at freeway merging areas. The proposed model adopts a non-cooperative decision-making approach involving two players: the driver of the mainline vehicle and the on-ramp vehicle. The payoff function, encompassing both efficiency-based and safety-based payoffs, is asymmetrically designed to evaluate safety loss and efficiency gain, adhering to the principles of prospect theory. Specifically, an S-shaped value function is formulated to assess the payoffs, and a first-best weighting function considering loss aversion is established within the framework of probability perception. A bilevel optimization approach is employed to estimate the model parameters. The upper level entails a nonlinear programming problem aimed at minimizing the squared difference between observed and predicted strategies, while the lower-level programming seeks the Nash equilibrium solution. Vehicle trajectory data is utilized for model calibration and validation purposes. Validation on the US-101 and I-80 datasets shows that the proposed model achieves detection rates of 92.14% and 92.34%, respectively. The corresponding validation errors are also reported using MAE and RMSE, rather than treating MAE as classification accuracy. Comparative results against representative game-theoretic mandatory lane-changing models further indicate that incorporating prospect-theory-based loss aversion improves both behavioural interpretability and predictive performance.

## 1. Introduction

Mandatory lane changes are a prevalent form of lane change observed in merging zones, primarily aimed at achieving the desired lane position. On one hand, they can alleviate congestion pressure for vehicles stuck behind slow-moving vehicles or platoons [1]. On the other hand, they can also create gaps that hinder the progress of following vehicles in the receiving lane, potentially leading to stop-and-go oscillations or even traffic breakdowns [2–5]. Considering the heightened disruption caused by their mandatory and forceful nature [6], this study focuses on modelling mandatory lane-changing behaviour at freeway on-ramps.

As a consequence, subsequent research has placed significant emphasis on understanding the detrimental effects of lane changes on traffic safety and flow when modelling lane-changing behaviours. The majority of existing lane-changing models are rule-based [7–9], often overlooking the stochastic and uncertain nature of driver behaviour. Conversely, some models utilize practical utility-based approaches to capture drivers’ decision-making processes [10–12]. However, these models do not explicitly consider the interactions among drivers and cognitive decision-making features.

Therefore, the game-theoretic method has gained widespread application in modelling lane-changing decisions [13–15] to address the intricate interplay among conflicting drivers. The lane-changing game typically considers lane-changing necessity, desirability, safety, etc. The utility of each driver depends not only on their actions but also on the actions of other drivers. This allows the game-theoretic method to describe drivers’ interactive behaviour and analyse the driving action mechanism during lane changes.

Nash Equilibrium (NE) is the most common assumption in the non-cooperative game solutions of lane-changing manoeuvres [16], where each driver lacks any incentive to change their initial strategy. Previous studies include models calibrated against data from observed field interactions [13,17], models with arbitrarily specified incentives for choices, and purely theoretical models. Diverse payoffs have been developed to capture drivers’ expectations, primarily encompassing efficiency-based and safety-based payoffs [4,15]. Nonetheless, variations in units and improper weighting coefficients attributed to diverse motivations and risk appetites in payoff functions frequently result in unrealistic equilibrium outcomes [15,17,18].

To address the aforementioned challenges in accurately modelling drivers’ lane-changing interactions and individual risk appetites, this study employs the framework of prospect theory [19] to formulate the payoff function. Efficiency-based and safety-based payoffs are defined as efficiency gains and safety losses, and compared to equivalent efficiency gains, safety losses elicit a heightened level of sensitivity in driver responses. The payoff function is thus founded on two fundamental elements: an adaptive value function and a probability weighting function. The former includes loss aversion and a range of risk attitudes within the domains of efficiency gains and safety losses, while the latter addresses systematic biases in probability perception. Consequently, it can accurately depict the real behaviour of drivers during mandatory lane changes in risky situations. This study presents the following contributions:

(1) This study develops a game-theoretic model for mandatory lane-changing behaviour that explicitly incorporates drivers’ risk appetites, which offers a new perspective on traffic flow dynamics in merging zones.
(2) The introduced prospect theory-based payoff function asymmetrically evaluates safety losses and efficiency gains, which provides a more accurate representation of drivers’ risk-sensitive decision-making in mandatory lane changes.
(3) The proposed bilevel optimization approach accurately calibrates the game model parameters, which offers insights into the intricate decision-making processes of drivers during mandatory lane changes under risk.

The remainder of the paper is structured as follows: Section 2 provides a concise overview of key applications of game theory in transportation engineering, accompanied by a review of critical lane-changing models. Section 3 introduces a methodology for mandatory lane-changing games. Section 4 delves into data sources, processing, and empirical evidence of strategies. Section 5 outlines the model calibration and validation, while also comparing its performance to other models. Section 6 addresses issues and presents the primary findings.

## 2. Literature review

Most of the lane-changing decision models in the literature are grounded in lane-changing rules. Gipps (1986) [7] was among the early pioneers who introduced a deterministic, rule-based structure for lane-changing decisions, aiming to ensure consistent vehicle behaviour in traffic simulations across various urban lane-changing scenarios. Drivers make lane change decisions based on sequential deterministic rules. Building upon Gipps’ model, Yang and Koutsopoulos (1996) [20] developed a lane-changing model tailored for freeways, distinguishing between mandatory and discretionary lane changes. Subsequently, several rule-based models [8,21] emerged to address specific limitations, including the requirement of safe conditions to initiate a lane change.

Ahmed et al. (1996) [22] introduced the utility-based lane-changing model, which focuses on individual vehicles and assesses lane-changing demand through lane utility evaluation. Ahmed (1999) [10] further extended this model to address heavily congested traffic, employing a three-step approach to model lane-changing manoeuvres. The existing literature offers a multitude of extensions and enhancements to utility-based models [11,23,24]. In addition to these widely adopted methods, researchers have explored alternative approaches to model lane-changing decision behaviour, including probabilistic methods [25,26], hazard-based models [27,28], and artificial intelligence techniques [29,30].

A game theory-based approach is also widely used in traffic modelling [31,32]. For lane-changing behaviour, Kita (1999) [13] formulated a merging model rooted in the Nash Equilibrium (NE) solution, treating the lane-changing decision as a non-zero-sum non-cooperative game between two players. Building on this foundation, researchers have improved other lane-changing decision models from various perspectives [15,17,18,33,34], refining actions and payoffs and incorporating various environmental factors. Furthermore, solutions such as social optimality and quantal response equilibrium are also employed in lane-changing modelling [35–38].

In summary, the majority of the approaches mentioned earlier, except for game theory, exclusively focus on the decision of the lane-changing vehicle while overlooking the corresponding response of the opponent vehicle. Neglecting the decisions of any participant could result in impractical predictions. Furthermore, a comprehensive literature review has identified several issues, most notably the failure to account for loss aversion concerning safety losses and efficiency gains in existing game theoretic models for mandatory lane changes. Addressing these issues is crucial for developing more realistic and behaviourally accurate mandatory lane-changing models.

Recent data-driven lane-changing models have shown strong predictive ability by learning nonlinear behavioural patterns directly from trajectory data. However, their internal decision mechanisms are often less transparent, and their generalisation can be affected by changes in traffic environment, vehicle composition, and driving style. In contrast, the present model explicitly formulates the interaction between the on-ramp vehicle and the mainline vehicle as a strategic decision process and provides behavioural interpretations through efficiency gain, safety loss, probability weighting, and loss aversion. Therefore, the proposed approach should be viewed as an interpretable behavioural complement to data-driven prediction models rather than a replacement for them.

## 3. Methodology

### 3.1 Mandatory lane-changing game

**Fig 1:**
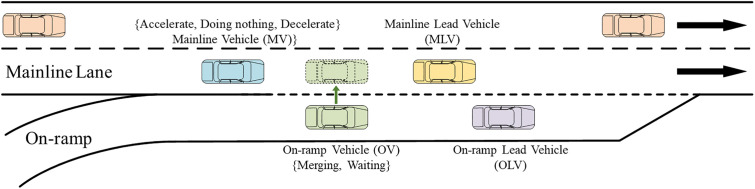
The schematic of a typical mandatory lane-changing manoeuvre.

In the context of mandatory lane changes, drivers rely on various information gathered by observing the vehicles around them to make informed decisions [39,40]. This decision-making process determines whether drivers should change lanes to reach their intended positions, thus improving the safety and reliability of mandatory lane changes. Drawing from game-theoretic research [13,35,41], the decision interaction is modelled as a non-zero-sum non-cooperative game involving drivers of the mainline vehicle (MV) and the on-ramp vehicle (OV). Fig 1 illustrates the schematic of a standard decision-making game process for mandatory lane changes at freeway on-ramps.

In the mandatory lane-changing game, MV has three pure strategies: “Accelerate”, “Do nothing”, and “Decelerate”, while OV has two pure strategies: “Merging” and “Waiting”. The choice of strategy for MV (“Accelerate”, “Do nothing”, or “Decelerate”) depends on its acceleration (a). The strategy of the mainline vehicle is classified according to its longitudinal acceleration. Following the steady-state regime definition of Ozaki (1993) [42] and its subsequent use in mandatory lane-changing studies (Ali et al., 2019, 2021, 2023) [35,43,44], a small dead-band of ±0.05g is used to distinguish active acceleration/deceleration from near-steady motion. Specifically, the strategy is classified as acceleration when a>0.05g, doing nothing when −0.05g≤a≤0.05g, and deceleration when a<−0.05g. This threshold is used as a behavioural classification rule rather than as a calibrated parameter. It also reduces the risk that small acceleration fluctuations caused by trajectory differentiation noise are misinterpreted as intentional manoeuvres. The “Merging” and “Waiting” strategies of OV depend on whether it attempts to merge into the gap in front of the MV. Table 1 presents the structure of the game for mandatory lane-changing decisions.

**Table 1:** Mandatory lane-changing decision game in standard form.

Payoff	On-ramp Vehicle (OV)	
Mainline Vehicle (MV)	Merging ($O_{1}$)	Waiting ($O_{2}$)
Accelerate ($M_{\text{1}}$)	$u_{OV}\left(O_{\text{1}},M_{\text{1}}\right)$,$u_{MV}\left(O_{\text{1}},M_{\text{1}}\right)$	$u_{OV}\left(O_{\text{2}},M_{\text{1}}\right)$,$u_{MV}\left(O_{\text{2}},M_{\text{1}}\right)$
Doing nothing ($M_{\text{2}}$)	$u_{OV}\left(O_{\text{1}},M_{\text{2}}\right)$,$u_{MV}\left(O_{\text{1}},M_{\text{2}}\right)$	$u_{OV}\left(O_{\text{2}},M_{\text{2}}\right)$,$u_{MV}\left(O_{\text{2}},M_{\text{2}}\right)$
Decelerate ($M_{\text{3}}$)	$u_{OV}\left(O_{\text{1}},M_{\text{3}}\right)$,$u_{MV}\left(O_{\text{1}},M_{\text{3}}\right)$	$u_{OV}\left(O_{\text{2}},M_{\text{3}}\right)$,$u_{MV}\left(O_{\text{2}},M_{\text{3}}\right)$

Note: $u_{OV}\left(O_{i},M_{j}\right)$: OV’s payoff for when OV adopts strategy $O_{i}$ and MV adopts strategy $M_{j}$; $u_{MV}\left(O_{i},M_{j}\right)$: MV’s payoff when OV adopts strategy $O_{i}$ and MV adopts strategy $M_{j}$, i=1,2, j=1,2,3.

Similar to other game-theoretic models, the lane-changing game model is based on several underlying assumptions, including: (a) both the OV and MV adhere to a car-following model during the lane change; (b) OV and MV reach an equilibrium state during the lane change process; (c) OV and MV are able to accurately calculate their respective payoffs in the lane-changing game; and (d) the distance between OV and MV does not exceed 60 meters.

### 3.2 Payoff function

The primary aim of this study is to model mandatory lane-changing decision behaviour within a comprehensive framework that accounts for drivers’ expectations. These expectations are influenced by various driver characteristics such as background, gender, age, among others, as drivers typically assign different values to different factors. However, a common objective among drivers is to minimize travel time while also being cognizant of potential risks or losses [36]. In this context, “efficiency gain” refers to the reduction in travel time, while “safety loss” pertains to the Time-To-Collision (TTC) between conflicting vehicles under different strategies. To capture these aspects comprehensively, the payoff function is formulated using prospect theory.

In this study, risk appetite is used as an aggregate behavioural construct describing how a driver trades efficiency gain against safety loss during a mandatory lane-changing interaction. It is not represented by a single parameter. Instead, it is operationalised through the curvature of the value function, the asymmetric sensitivity to safety losses, and the nonlinear relative weighting of gain and loss components. The curvature parameter α reflects diminishing sensitivity, whereas the ratio $\beta_{\text{L}}/\beta_{\text{G}}$ reflects loss aversion, namely the stronger impact of safety loss than an equivalent efficiency gain. The weighting function further describes how the relative importance of efficiency gain and safety loss is perceived by the driver.

#### 3.2.1 Efficiency gain.

The extra time spent by various strategy profiles is quantified as the travel time difference ΔT [36,45], and it can be calculated as:


$$\begin{array}{c} \begin{array}{c} \Delta T^{N}\left(O_{i},M_{j}\right)=T^{N}\left(O_{i},M_{j}\right)-T_{min}^{N} \end{array} \end{array}$$ (1)

where $\Delta T^{N}\left(O_{i},M_{j}\right)$: Travel time difference of player N under the strategy profile $\left(O_{i},M_{j}\right)$; $T^{N}\left(O_{i},M_{j}\right)$: Travel time of player N under the strategy profile $\left(O_{i},M_{j}\right)$; $T_{min}^{N}$: Minimum travel time of player N.

The calculation of travel time involves constructing parallel worlds where the car-following model is utilized to evaluate the potential gains and losses linked to drivers’ actions. This approach builds upon the car-following model grounded in prospect theory, initially introduced by Hamdar et al. (2008) [46]. Talebpour et al. (2011) [47] further extended the applicability of this model to encompass both congested and uncongested traffic scenarios. The value functions $U_{PT}^{C}\left(a_{n}\right)$ and $U_{PT}^{UC}\left(a_{n}\right)$ are defined as follows:


$$\begin{array}{c} \begin{array}{c} U_{PT}^{C}\left(a_{n}\right)=\frac{\left(\omega_{n}^{C}+(\text{1}-\omega_{n}^{C})*\left(\text{tanh}\left(\frac{a_{n}}{a_{\text{0}}}\right)+\text{1}\right)\right)}{\text{2}}*{\left[\left|\frac{a_{n}}{a_{\text{0}}}\right|\right]}^{\tau^{C}}*sgn\left(a_{n}\right) \end{array} \end{array}$$ (2)

$$\begin{array}{c} \begin{array}{c} U_{PT}^{UC}\left(a_{n}\right)=\frac{\left(\omega_{n}^{UC}+(\text{1}-\omega_{n}^{UC})*\left(\text{tanh}\left(\frac{a_{n}}{a_{\text{0}}}\right)+\text{1}\right)\right)}{\text{2}}*{\left(\frac{\left(\frac{a_{n}}{a_{\text{0}}}\right)}{\text{1}+{\left(\frac{a_{n}}{a_{\text{0}}}\right)}^{\text{2}}}\right)}^{\tau^{UC}} \end{array} \end{array}$$ (3)

where $U_{PT}^{C}\left(a_{n}\right)$ and $U_{PT}^{UC}\left(a_{n}\right)$: The value function for the congested and uncongested traffic conditions; $a_{n}$: Acceleration choice for driver n; $a_{\text{0}}$: Used for normalizing the acceleration. $\omega_{n}^{C}$, $\;\omega_{n}^{UC}$, $\tau^{C}>\text{0}$, and $\tau^{UC}>\text{0}$: Parameters to be estimated.

Drivers employ the corresponding value function based on their perception of surrounding traffic condition. The expected value function $U_{PT}\left(a_{n}\right)$ is calculated by using the following binary probabilistic regime selection mechanism:


$$\begin{array}{c} \begin{array}{c} U_{PT}\left(a_{n}\right)=P^{C}*U_{PT}^{C}\left(a_{n}\right)+P^{UC}*U_{PT}^{UC}\left(a_{n}\right) \end{array} \end{array}$$ (4)

where $P^{C}$ and $P^{UC}$: The respective probabilities of driving in the congested and uncongested traffic conditions.

Total utility function $U\left(a_{n}\right)$ of acceleration can be formulated as follows:


$$\begin{array}{c} \begin{array}{c} U\left(a_{n}\right)=\left(\text{1}-p_{n,i}\right)U_{PT}\left(a_{n}\right)-p_{n,i}\omega_{c}k(v,\Delta v) \end{array} \end{array}$$ (5)

where $p_{n,i}$: The probability of a crash at the end of the duration;$\;\omega_{c}$: A weighting factor; k(v,Δv): The crash seriousness term.

To reflect the stochastic response adopted by the drivers, the acceleration of vehicle is retrieved from the following probability density function:


$$\begin{array}{c} \begin{array}{c} f\left(a_{n}\right)=\left\{ \begin{array}{cc} \frac{\text{exp}[(\vartheta *U\left(a_{n}\right))]}{\int_{a_{min}}^{a_{max}}\text{exp}[(\vartheta *U\left(a^{\prime }\right))]da^{\prime }} & a_{min}{\leq a}_{n}\leq a_{max}\\ \text{0} & otherwise \end{array}\right.\; \end{array} \end{array}$$ (6)

where ϑ: A free parameter (ϑ>0) that reflects the sensitivity of choice to the utility $U\left(a_{n}\right)$; $a_{max}$ and $a_{min}$: Maximum and minimum acceleration.

Therefore, efficiency gain of OV and MV under various strategy profiles can be represented using the following formula:


$$\begin{array}{c} \begin{array}{c} \left\{ \begin{array}{c} @lu_{EG}^{OV}\left(O_{i},M_{j}\right)=\text{exp}\left[a_{ij}^{\text{1}}/\Delta T^{OV}\left(O_{i},M_{j}\right)\right]\\ u_{EG}^{MV}\left(O_{i},M_{j}\right)=\text{exp}\left[b_{ij}^{\text{1}}/\Delta T^{MV}\left(O_{i},M_{j}\right)\right] \end{array}\right.\; \end{array} \end{array}$$ (7)

where $a_{ij}^{k}$, and $b_{ij}^{k}$: Parameters to be estimated, i=1,2, j=1,2,3,k=1,2,3.

#### 3.2.2 Safety loss.

Safety loss is quantified as TTC between vehicles involved in conflicting scenarios under various strategy profiles. Let’s first consider OV. When it changes lanes, its safety loss is determined by ${TTC}_{MV}^{OV}$ between MV and OV, and ${TTC}_{OV}^{MLV}$ between OV and the mainline lead vehicle (MLV).


$$\begin{array}{c} \begin{array}{c} u_{SL}^{OV}\left(O_{\text{1}},M_{j}\right)=\text{exp}\left(\frac{a_{\text{1}j}^{\text{2}}}{{TTC}_{MV}^{OV}}\right)+\text{exp}(\frac{a_{\text{1}j}^{\text{3}}}{{TTC}_{OV}^{MLV}}) \end{array} \end{array}$$ (8)

However, if OV opts to wait in the acceleration lane, its safety loss is calculated as ${TTC}_{OV}^{OLV}$ between OV and on-ramp lead vehicle (OLV).


$$\begin{array}{c} \begin{array}{c} u_{SL}^{OV}\left(O_{\text{2}},M_{j}\right)=\text{exp}\left(\frac{a_{\text{2}j}^{\text{2}}}{{TTC}_{OV}^{OLV}}\right) \end{array} \end{array}$$ (9)

Now, let’s turn our attention to MV. If OV chooses to change lanes, we account for the potential conflict between MV and OV in the safety loss, expressed as ${TTC}_{MV}^{OV}$. However, if OV opts to wait in the acceleration lane, the safety loss is represented by ${TTC}_{MV}^{MLV}$ between MV and MLV.


$$\begin{array}{c} \begin{array}{c} u_{SL}^{MV}\left(O_{\text{1}},M_{j}\right)=\text{exp}\left(\frac{b_{\text{1}j}^{\text{2}}}{{TTC}_{MV}^{OV}}\right),\;j=\text{1},\text{2},\text{3} \end{array} \end{array}$$ (10)

$$\begin{array}{c} \begin{array}{c} u_{SL}^{MV}\left(O_{\text{2}},M_{j}\right)=\text{exp}\left(\frac{b_{\text{2}j}^{\text{2}}}{{TTC}_{MV}^{MLV}}\right)\;,\;j=\text{1},\text{2},\text{3} \end{array} \end{array}$$ (11)

Because the efficiency-gain and safety-loss components contain inverse travel-time and inverse-TTC terms, numerical safeguards were applied before evaluating the payoff functions. Travel-time differences and TTC values were first checked for missing or non-physical values. If the required leader-follower relationship could not be reliably reconstructed, the corresponding interaction was excluded. For valid interactions, the denominators were bounded below by small positive constants. Extremely large TTC values, which indicate practically non-conflicting situations, were capped at 60 s to prevent unbounded differences from dominating the payoff calculation. These treatments were applied consistently to all candidate strategy profiles and all comparison models.

#### 3.2.3 Probability weighting.

Prospect theory, pioneered by Kahneman (1979) [19] and further refined by Tversky and Kahneman (1992) [48], stands out as one of the most successful descriptive theories concerning decision-making under risk [49–51]. It hinges on two fundamental components: an adaptive value function and a probability weighting function. The former encapsulates concepts such as loss aversion and varying risk attitudes towards gains and losses, while the latter addresses systematic distortions in probability perception. Leveraging prospect theory can aid in constructing a payoff function that effectively integrates efficiency gains and safety losses in the lane-changing game.

**Fig 2:**
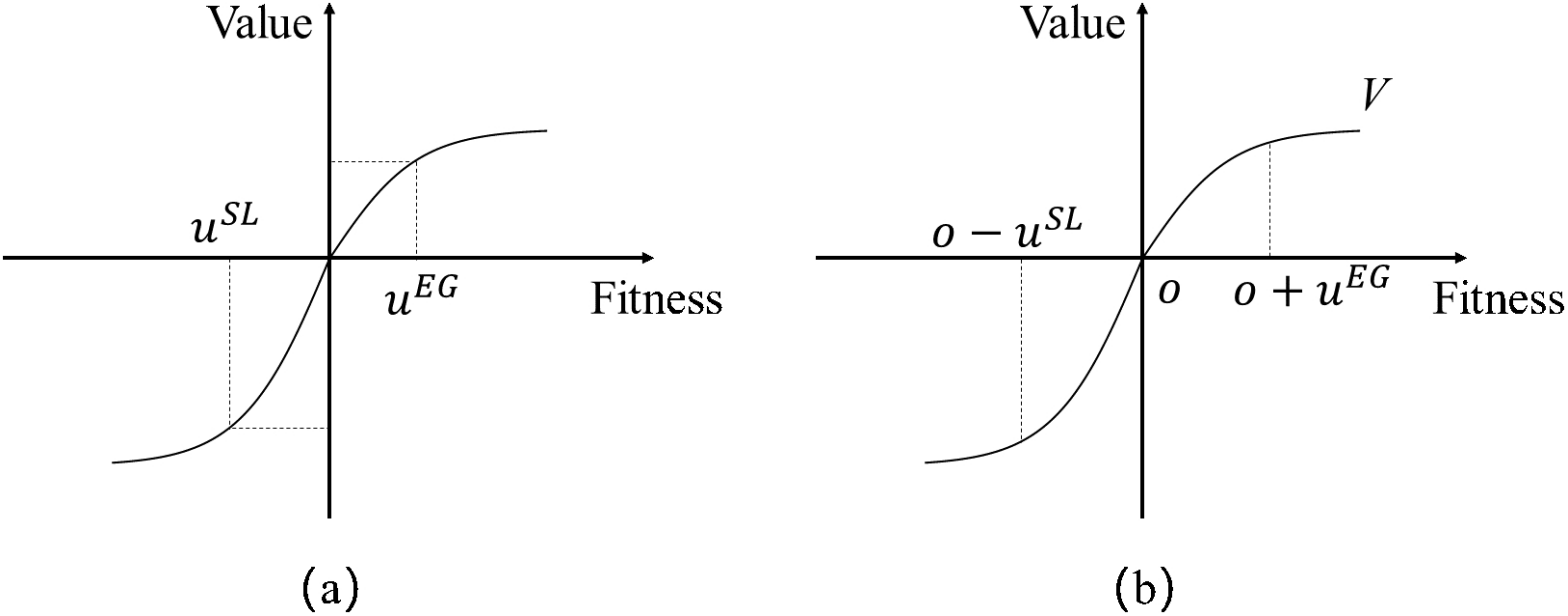
The value function: (a) Typical value function, (b) Transformed value function.

Building upon the framework proposed by Herold and Netzer (2023) [52], and referring to a typical value function illustrated in Fig 2(a), This study posits that the decision-maker employs an S-shaped value function V:ℝ→ℝ to assess gains and losses with respect to o, where V(o)=0 as portrayed in Fig 2(b) In particular, This study defines $v_{G}:{\mathbb{R}}_{+}\to {\mathbb{R}}_{+}$ as $v_{G}(u_{EG})=V\left(o+u_{EG}\right)$ for $u_{EG}>\text{0}$, and $v_{L}:{\mathbb{R}}_{+}\to {\mathbb{R}}_{+}$ as $v_{L}\left(u_{SL}\right)=-V\left(o-u_{SL}\right)$ for $u_{SL}>\text{0}$. This study posits that both $v_{G}$ and $v_{L}$ exhibit properties of continuous differentiability, strict monotonousness, strict concavity, and unboundedness.

In the context of probability perception, this study introduces $\eta :{\mathbb{R}}_{+}\to {\mathbb{R}}_{+}$, a measurable weighting function that generates a perceived relative probability η(φ) based on the true relative probability $\varphi =\frac{\gamma }{\left(\text{1}-\gamma \right)}$, where γ and 1−γ represent the weights of efficiency gains and safety losses, respectively. Consequently, the decision-making driver perceives the actual gain weight γ as $\frac{\eta (\varphi )}{\left(\text{1}+\eta (\varphi )\right)}$ and the loss weight 1−γ as $\frac{\text{1}}{\left(\text{1}+\eta (\varphi )\right)}$. Keeping these concepts in mind, the overall payoff function attributed to a prospect can be


$$\begin{array}{c} \begin{array}{c} u_{\eta }\left(\varphi ,u_{EG},u_{SL}\right)=\left(\frac{\eta (\varphi )}{\text{1}+\eta (\varphi )}\right)v_{G}\left(u_{EG}\right)-\left(\frac{\text{1}}{\text{1}+\eta (\varphi )}\right)v_{L}\left(u_{SL}\right) \end{array} \end{array}$$ (12)

Define $\pi_{G}:[\text{0},\text{1}]\to [\text{0},\text{1}]$ as a function that associates the gain weight γ with a gain decision weight $\pi_{G}(\gamma )$, and likewise, define $\pi_{L}:[\text{0},\text{1}]\to [\text{0},\text{1}]$ as a function that links the loss weight 1−γ to a loss decision weight $\pi_{L}(\text{1}-\gamma )$. The absolute weighting functions $\pi_{G}$ and $\pi_{L}$ are connected to the relative weighting function η in the following manner:


$$\begin{array}{c} \begin{array}{c} \eta_{\left(\pi_{G},\pi_{L}\right)}(q)=\frac{\pi_{G}\left(\frac{\varphi }{\text{1}+\varphi }\right)}{\pi_{L}\left(\frac{\text{1}}{\text{1}+\varphi }\right)} \end{array} \end{array}$$ (13)

**Fig 3:**
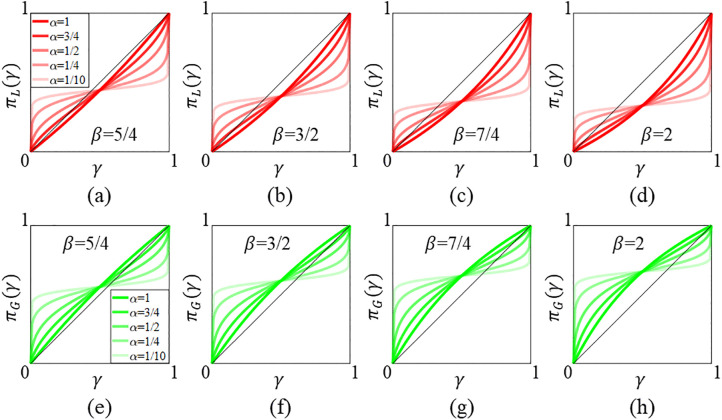
Non-reflective weighting in various loss-aversion case.


Construct value functions $v_{G}\left(u_{EG}\right)=\beta_{G}{u_{EG}}^{\alpha }$ and $v_{L}\left(u_{SL}\right)=\beta_{L}{u_{SL}}^{\alpha }$ with $\beta_{G},\beta_{L},\alpha >\text{0}$, taking into account $\eta^{FB}(\varphi )=(\beta_{L}/\beta_{G})\varphi^{\alpha }$ as a first-best weighting function. In the case of loss aversion ($\beta_{L}>\beta_{G}$), $\pi_{G}$ and $\pi_{L}$ can be computed as follows, which are illustrated in Fig 3 for the case of $\beta =\beta_{L}/\beta_{G}\in \{\text{5}/\text{4},\;\text{3}/\text{2},\text{7}/\text{4},\text{2}\}$ and α∈{1,3/4,1/2,1/4,1/10}:

$$\begin{array}{c} \begin{array}{c} @l\left\{ \begin{array}{c} @l\pi_{G}^{\eta^{FB}}(\gamma )=\frac{\left(\frac{\beta_{L}}{\beta_{G}}\right)\gamma^{\alpha }}{\left(\frac{\beta_{L}}{\beta_{G}}\right)\gamma^{\alpha }+(\text{1}-\gamma {)}^{\alpha }}\\ \pi_{L}^{\eta^{FB}}(\gamma )=\frac{\left(\frac{\beta_{G}}{\beta_{L}}\right)\gamma^{\alpha }}{\left(\frac{\beta_{G}}{\beta_{L}}\right)\gamma^{\alpha }+(\text{1}-\gamma {)}^{\alpha }} \end{array}\right.\; \end{array} \end{array}$$ (14)

## 4. Mandatory lane-changing data

This section provides the essential mandatory lane-changing data required for model calibration and validation, sourced from the celebrated Next Generation Simulation (NGSIM) datasets.

The unit of analysis in this study is a pairwise mandatory lane-changing interaction event rather than an entire vehicle trajectory. For each mandatory lane-changing interaction, the on-ramp vehicle (OV) was paired with the mainline vehicle (MV) immediately behind the target gap that the OV attempted to enter or evaluated. The vehicle ahead of this gap was defined as the mainline lead vehicle (MLV), and the vehicle ahead of the OV in the on-ramp or auxiliary lane was defined as the on-ramp lead vehicle (OLV). An interaction event refers to one OV-MV decision instance within the 60 m longitudinal interaction range. The starting moment of an event was defined as the first time at which the target gap could be identified and the OV began to approach or evaluate that gap. The ending moment was defined as the decision moment, namely the lane-boundary crossing time for a merging event or the last feasible decision time before the OV continued to wait and the original target gap was no longer selected. One physical vehicle may appear in more than one observation only when it is involved in different pairwise interaction events; however, repeated records for the same OV-MV pair at the same decision moment were removed.

### 4.1 US-101 data

The vehicle trajectory data used in this study were gathered at 7:50 a.m. on June 15th, from the southbound US-101 in Los Angeles, CA. The study area spanned approximately 640 meters (2,100 feet) and included five mainline lanes along the section. Additionally, an auxiliary lane was present along a segment of the corridor, extending from the on-ramp at Ventura Boulevard to the off-ramp at Cahuenga Boulevard.

In this study, the decision of OV was directly inferred from the trajectory data, while the decision of MV was initially formulated based on theoretical knowledge and subsequently validated using field observations and the Bottom-Up segmentation algorithm [41]. Strategies derived from field observations were formulated based on slopes calculated using the Bottom-Up algorithm. The merging or waiting point in OV’s trajectory was used to determine the corresponding point in MV’s trajectory. At the corresponding time, if the slope was positive, MV’s strategy was classified as acceleration; if the slope was negative, MV’s strategy was categorized as deceleration. If the slope fell within the range of 0.05g to −0.05g, MV’s strategy was classified as maintaining the current speed.

The US-101 data yielded a total of 1,571 observations, including 637 instances of ‘Merging’ actions and 934 instances of ‘Waiting’ actions for OV. Additionally, there were 334 instances of ‘Accelerate’ actions, 851 instances of ‘Doing nothing’ actions, and 386 instances of ‘Decelerate’ actions for MV. Table 2 provides empirical evidence of the strategies extracted from the US-101 data.

**Table 2:** Empirical evidence of strategies extracted from NGSIM data.

Strategy profile	US-101		I-80	
	Count	Percentage	Count	Percentage
S-1	158	10.06%	145	9.25%
S-2	314	19.99%	327	20.85%
S-3	165	10.50%	126	8.04%
S-4	176	11.20%	103	6.57%
S-5	537	34.18%	615	39.22%
S-6	221	14.07%	252	16.07%

S-1: $\left(O_{\text{1}},M_{\text{1}}\right)$; S-2: $\left(O_{\text{1}},M_{\text{2}}\right)$; S-3: $\left(O_{\text{1}},M_{\text{3}}\right)$; S-4: $\left(O_{\text{2}},M_{\text{1}}\right)$; S-5: $\left(O_{\text{2}},M_{\text{2}}\right)$; S-6: $\left(O_{\text{2}},M_{\text{3}}\right)$.

### 4.2 I-80 data

The vehicle trajectory data utilized in this study were collected at 4:00 p.m. on April 13th from the eastbound lanes of I–80 in the San Francisco Bay area, specifically Emeryville, CA. The study area encompassed approximately 500 meters (1,640 feet) in length and included six freeway lanes, with an on-ramp located within the study area. A total of 1,568 observations were extracted from the I–80 data, comprising 598 instances of “Merging” actions and 970 instances of “Waiting” actions for OV, as well as 248 instances of “Accelerate” actions, 942 instances of “Doing nothing” actions, and 378 instances of “Decelerate” actions for MV. Table 2 provides empirical evidence of the strategies obtained from the I–80 data.

Before extracting interaction events, the original NGSIM trajectories were reconstructed and smoothed to reduce unrealistic position jumps, speed fluctuations, and acceleration noise. This preprocessing step is necessary because velocity and acceleration estimates obtained by differentiating raw NGSIM position records may be noisy (Thiemann et al., 2008). The reconstruction method follows Montanino and Punzo (2015) [53], and the event extraction procedure follows Ali et al. (2023) [44]. The Bottom-Up segmentation algorithm was then applied to identify approximately homogeneous motion segments. The MV’s response strategy was classified using the smoothed acceleration segment that contains the OV’s merging/waiting decision moment. The data were randomly split, with seventy percent allocated for calibration and the remaining thirty percent for validation. Additionally, the data were randomly divided into ten subsets for cross-validation testing.

## 5. Model calibration and validation

### 5.1 Model calibration

#### 5.1.1 Calibration approach.

The calibration process is designed to identify a set of model parameters that minimize the disparity between observed and predicted merging decisions. To achieve this, this study utilizes the calibration framework proposed by Liu et al. (2007) [17] and Ali et al. (2019) [35]. Within this framework, the parameters are estimated by solving a bilevel programming problem. The upper level of this problem entails a non-linear programming task aimed at minimizing the squared difference between the observed and predicted strategies. This is expressed as follows:


$$\begin{array}{c} \begin{array}{c} \text{min}\sum_{i=\text{1}}^{n}\left[{\left(P_{i}-{\hat{P}}_{i}\left(y_{i}\right)\right)}^{\text{2}}+{\left(Q_{i}-{\hat{Q}}_{i}\left(z_{i}\right)\right)}^{\text{2}}\right] \end{array} \end{array}$$ (15)

where i: An index of an observation; $P_{i}$: The observed strategy of OV; ${\hat{P}}_{i}$: The predicted strategy for OV;$\;Q_{i}$: The observed strategy of MV; ${\hat{Q}}_{i}$: The predicted strategy for MV;$\;y_{i}$: The probability of OV’s choice; $z_{i}$: The probabilities of MV’s choice.

The lower-level programming seeks the NE solution, defined as follows:


$$\begin{array}{c} \begin{array}{c} \left\{ \begin{array}{c} @lu_{OV}\left(O^{*},M^{*}\right)\geq u_{OV}\left(O,M^{*}\right)\\ u_{MV}\left(O^{*},M^{*}\right)\geq u_{MV}\left(O^{*},M\right) \end{array}\right.\; \end{array} \end{array}$$ (16)

**Fig 4:**
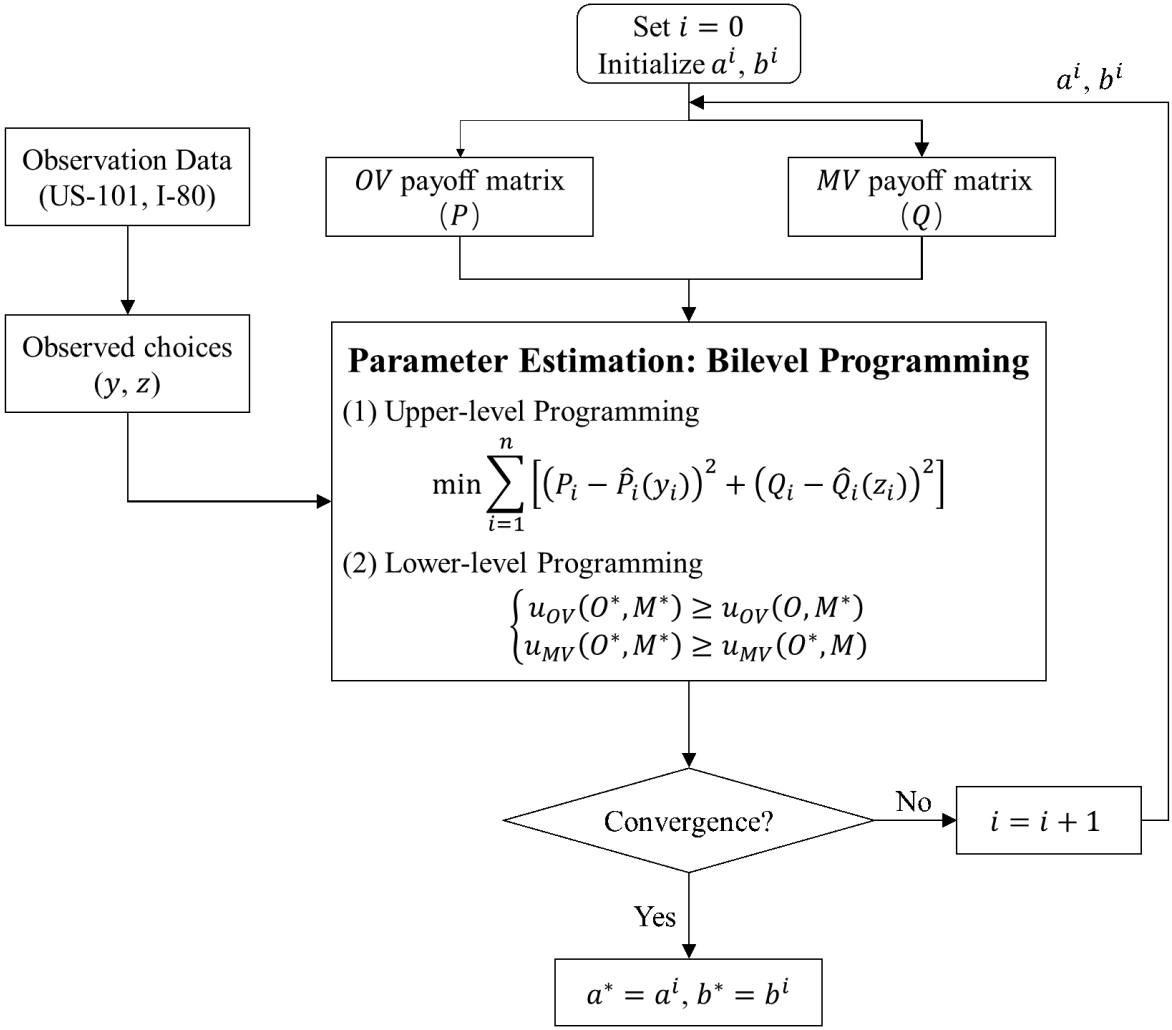
Calibration framework for bilevel programming.

The bilevel programming problem is solved with reference to method proposed by Ali et al. (2019) [35], as shown in Fig 4.

For each observation, the six pure strategy profiles in the 2 x 3 game matrix were exhaustively evaluated. A strategy profile was identified as a Nash equilibrium when both the OV and MV best-response inequalities were satisfied. Under the continuous payoff values and a numerical tolerance of 10^−8^, no exact multiple-equilibrium cases were observed in the analysed samples. For reproducibility, if multiple equilibria occur in future applications, the model selects the equilibrium with the largest sum of the two players’ normalised payoffs; if a tie remains, the profile with the smaller total safety loss is selected; if the tie still remains, the less aggressive MV response is selected in the order of doing nothing, decelerating, and accelerating.

The bilevel optimisation was implemented in MATLAB. For each candidate parameter set, the lower-level game was solved by exhaustive enumeration of the six pure strategy profiles. The upper-level nonlinear least-squares problem was solved using a sequential quadratic programming routine with bound constraints. The payoff parameters a and b were constrained to be positive, with bounds [10^−3^, 5] to avoid degenerate or unbounded payoffs. The values of α and β were evaluated over the candidate grids. To reduce dependence on local optima, 20 random initial parameter vectors were tested for each dataset and parameter combination, and the solution with the smallest objective value was retained. The optimisation was terminated when the change in the objective value was below 10^−6^ or when the maximum number of 1000 iterations was reached.

#### 5.1.2 Calibration result.

In the case of the US-101 data, calibration involved 446 out of 637 merging events and 654 out of 934 waiting events. For the I-80 data, calibration utilized 419 out of 598 merging events and 679 out of 970 waiting events. The parameter estimates for our proposed model when applied to the US-101 data and the I-80 data are presented in Tables 3 and 4, respectively. The Mean Absolute Error (MAE) for calibration is computed using Equation (17). For the US-101 data, the MAE is 0.09, indicating that our model can precisely predict 91% of mandatory lane-changing decisions in the US-101 dataset. For the I-80 data, the MAE is 0.08, suggesting that our model can accurately predict 92% of mandatory lane-changing decisions in the I-80 dataset.

**Table 3:** Estimation result for parameters in the payoff function using the US-101 data.

Strategy profile	player	Parameter	Value	Parameter	Value	Parameter	Value
S-1	OV	$a_{\text{11}}^{\text{1}}$	0.948	$a_{\text{11}}^{\text{2}}$	2.244	$a_{\text{11}}^{\text{3}}$	1.594
S-2		$a_{\text{12}}^{\text{1}}$	2.156	$a_{\text{12}}^{\text{2}}$	2.561	$a_{\text{12}}^{\text{3}}$	1.154
S-3		$a_{\text{13}}^{\text{1}}$	3.159	$a_{\text{13}}^{\text{2}}$	1.503	$a_{\text{13}}^{\text{3}}$	2.484
S-4		$a_{\text{21}}^{\text{1}}$	2.894	$a_{\text{21}}^{\text{2}}$	2.945		
S-5		$a_{\text{22}}^{\text{1}}$	1.599	$a_{\text{22}}^{\text{2}}$	2.162		
S-6		$a_{\text{23}}^{\text{1}}$	1.355	$a_{\text{23}}^{\text{2}}$	3.081		
S-1	MV	$b_{\text{11}}^{\text{1}}$	2.101	$b_{\text{11}}^{\text{2}}$	1.070		
S-2		$b_{\text{12}}^{\text{1}}$	1.724	$b_{\text{12}}^{\text{2}}$	1.669		
S-3		$b_{\text{13}}^{\text{1}}$	1.301	$b_{\text{13}}^{\text{2}}$	2.727		
S-4		$b_{\text{21}}^{\text{1}}$	2.423	$b_{\text{21}}^{\text{2}}$	2.424		
S-5		$b_{\text{22}}^{\text{1}}$	1.749	$b_{\text{22}}^{\text{2}}$	2.023		
S-6		$b_{\text{23}}^{\text{1}}$	1.977	$b_{\text{23}}^{\text{2}}$	0.987		
MAE=0.09							

**Table 4:** Estimation result for parameters in the payoff function using the I-80 data.

Strategy profile	player	Parameter	Value	Parameter	Value	Parameter	Value
S-1	OV	$a_{\text{11}}^{\text{1}}$	1.727	$a_{\text{11}}^{\text{2}}$	2.915	$a_{\text{11}}^{\text{3}}$	1.458
S-2		$a_{\text{12}}^{\text{1}}$	3.047	$a_{\text{12}}^{\text{2}}$	1.656	$a_{\text{12}}^{\text{3}}$	1.524
S-3		$a_{\text{13}}^{\text{1}}$	3.058	$a_{\text{13}}^{\text{2}}$	0.869	$a_{\text{13}}^{\text{3}}$	2.421
S-4		$a_{\text{21}}^{\text{1}}$	2.726	$a_{\text{21}}^{\text{2}}$	2.38		
S-5		$a_{\text{22}}^{\text{1}}$	3.098	$a_{\text{22}}^{\text{2}}$	2.726		
S-6		$a_{\text{23}}^{\text{1}}$	2.471	$a_{\text{23}}^{\text{2}}$	1.606		
S-1	MV	$b_{\text{11}}^{\text{1}}$	2.638	$b_{\text{11}}^{\text{2}}$	3.165		
S-2		$b_{\text{12}}^{\text{1}}$	1.911	$b_{\text{12}}^{\text{2}}$	2.492		
S-3		$b_{\text{13}}^{\text{1}}$	0.898	$b_{\text{13}}^{\text{2}}$	2.827		
S-4		$b_{\text{21}}^{\text{1}}$	1.201	$b_{\text{21}}^{\text{2}}$	2.107		
S-5		$b_{\text{22}}^{\text{1}}$	3.197	$b_{\text{22}}^{\text{2}}$	2.401		
S-6		$b_{\text{23}}^{\text{1}}$	1.569	$b_{\text{23}}^{\text{2}}$	2.398		
MAE=0.08							


$$\begin{array}{c} \begin{array}{c} MAE=\frac{\text{1}}{n}\sum_{i=\text{1}}^{n}\left|x_{i}-{\hat{x}}_{i}\right| \end{array} \end{array}$$ (17)

where x represents the actual observation; $\hat{x}$ is the model predicted decision; n is the number of observations; *i* is an index of the observations.

### 5.2 Model validation

#### 5.2.1 Calibration metrics.

Model validation is crucial for assessing the predictive capability of the calibrated model using a validation dataset. In this section, this study evaluates the model’s performance in predicting lane-changing behaviour based on the calibration results. In addition to the MAE, this study also employs the Root Mean Square Error (RMSE) to validate the proposed model, following the methodology outlined in Liu et al. (2007) [17]. RMSE is calculated as follow:


$$\begin{array}{c} RMSE=\sqrt{\frac{\text{1}}{n}\sum_{i=\text{1}}^{n}{\left(x_{i}-{\hat{x}}_{i}\right)}^{\text{2}}} \end{array}$$ (18)

In addition to MAE and RMSE, the performance indicators highlighted by Zheng (2014) [54] used also include true positive (TP, cases where the prediction matches the observation); false positive (FP, cases where the prediction doesn’t match the observation); detection rate (DR, the percentage of correct prediction); false alarm rate (FAR, the percentage of wrong prediction); lane-changing time errors (TE, time difference between the prediction and the observation); and lane-changing location errors (LE, location difference between the prediction and the observation).


$$\begin{array}{c} \begin{array}{c} DR=TP/N \end{array} \end{array}$$ (19)

$$\begin{array}{c} \begin{array}{c} FAR=FP/N \end{array} \end{array}$$ (20)

$$\begin{array}{c} \begin{array}{c} TE=\left|T_{p}-T_{o}\right| \end{array} \end{array}$$ (21)

$$\begin{array}{c} \begin{array}{c} LE=\left|L_{p}-L_{o}\right| \end{array} \end{array}$$ (22)

where N represents the number of observations; $T_{p}$ is the predicted lane-changing time; $T_{o}$ is the observed lane-changing time; $L_{p}$ is the predicted lane-changing location; $L_{o}$ is the observed lane-changing location.

#### 5.2.2 Validation results.

**Fig 5:**
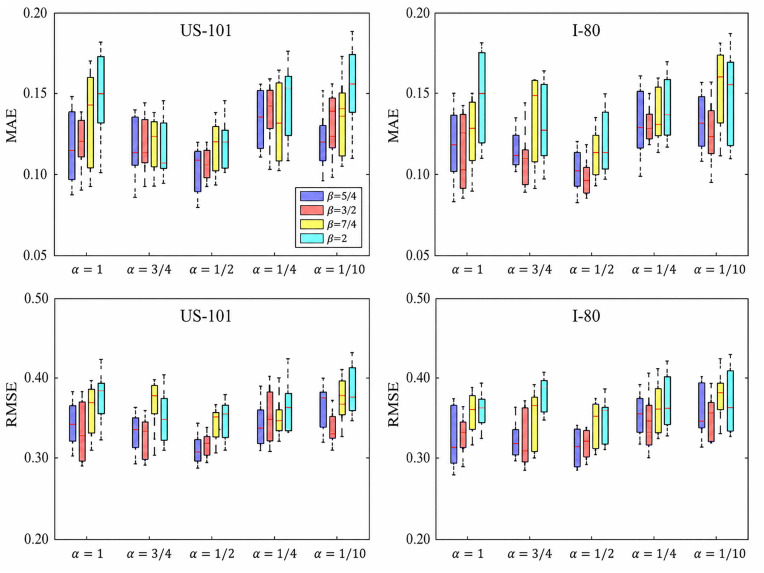
Validation results in MAE and RMSE.

Fig 5 illustrates the MAE and RMSE values across various scenarios using both the US-101 and I-80 data. When analysing the US-101 data, the mean MAE ranges from 0.11 to 0.16, while the mean RMSE varies from 0.31 to 0.38. On the other hand, when utilizing the I-80 data, the mean MAE ranges from 0.10 to 0.16, and the mean RMSE ranges from 0.30 to 0.39. These results indicate the model’s robust predictive capabilities and its ability to accurately forecast mandatory lane-changing behaviour.

The model effectively captures the underlying patterns and dynamics of drivers’ decision-making processes during lane-changing manoeuvres. Particularly noteworthy is the scenario with α=1/2 and β=5/4 using the US-101 data, where the minimum MAE is 0.08, achieving an impressive accuracy rate of 92%. This highlights the model’s robustness in replicating and predicting vehicle actions effectively. It suggests that drivers prioritize safety losses in the lane-changing game when making decisions, emphasizing the importance of safety considerations in their decision-making processes.

Table 5 displays the DR for various strategy profiles, denoted as S-1 to S-6, using the US-101 data. The DR values for each profile are as follows: 95.74%, 94.68%, 88.00%, 86.79%, 92.55%, and 92.42%. These high DR values indicate a strong alignment between predictions and observations for most scenarios. For the US-101 validation data, the proposed model correctly predicts 434 out of 471 interaction events, corresponding to an overall detection rate of 92.14%. Specifically, 178 merging events and 256 waiting events are correctly predicted. For the I-80 validation data, the model correctly predicts 434 out of 470 interaction events, corresponding to an overall detection rate of 92.34%, including 164 correctly predicted merging events and 270 correctly predicted waiting events. These findings underscore the effectiveness of our proposed model in predicting observed merging behaviour and its robust predictive capability for each strategy profile. This emphasizes the significant influence of risk perception and aversion on drivers’ decision-making. This underscores the importance of integrating psychological factors into modelling frameworks to achieve more accurate representations of real-world scenarios.

**Table 5:** Model validation results in the cases using the US-101 and I-80 data.

Data	US-101					I-80				
Strategy	N	TP	FP	DR (%)	FAR (%)	N	TP	FP	DR (%)	FAR (%)
S-1	47	45	2	95.74	4.26	43	41	2	95.35	4.65
S-2	94	89	5	94.68	5.32	98	92	6	93.88	6.12
S-3	50	44	6	88.00	12.00	38	31	7	81.58	18.42
S-4	53	46	7	86.79	13.21	31	28	3	90.32	9.68
S-5	161	149	12	92.55	7.45	184	173	11	94.02	5.98
S-6	66	61	5	92.42	7.58	76	69	7	90.79	9.21
Merging	191	178	13	93.19	6.81	179	164	15	91.62	8.38
Waiting	280	256	24	91.43	8.57	291	270	21	92.78	7.22
Total	471	434	37	92.14	7.86	470	434	36	92.34	7.66

The sensitivity results also provide behavioural insight. The parameter α controls the curvature of the value function; a smaller α and β implies stronger diminishing sensitivity to marginal changes in efficiency gain and safety loss. The ratio β reflects the degree of loss aversion, with larger values indicating a stronger behavioural weight assigned to safety loss relative to efficiency gain. The better-performing combinations, such as moderate α and β values, suggest that drivers in the analysed merging areas are neither purely linear payoff maximisers nor extremely loss-averse decision makers. Instead, their decisions are better described by moderate nonlinear sensitivity and moderate asymmetry between safety loss and efficiency gain. The relatively lower detection rates for S-3 and S-4 may be attributed to their lower sample shares and behavioural ambiguity. In S-3, OV merges while MV decelerates, which may reflect either cooperative yielding or forced merging under short gaps. In S-4, OV waits while MV accelerates, and this response can be influenced by anticipation, local speed recovery, and acceleration fluctuations around the threshold. These profiles are therefore more difficult to separate using discrete strategy labels.

**Fig 6:**
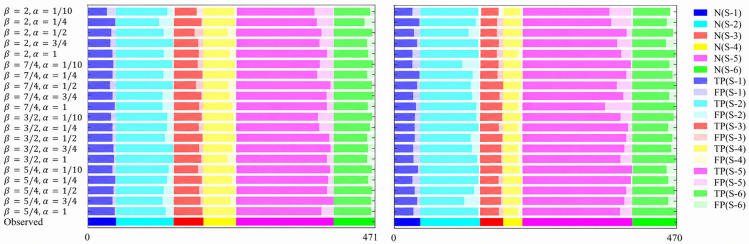
Validation results in TP and FP.

To demonstrate the model’s predictive capabilities across various scenarios, Fig 6 presents the mean TP and FP values obtained from 10 rounds of cross-validation testing with different α and β values, utilizing both US-101 and I-80 data. Despite fluctuations in the model’s predictive ability due to varying α and β values, the overall prediction accuracy remains consistently high. When using the US-101 data, the model achieves its highest prediction performance with α=3/4 and β=3/2, accurately predicting 437 interactions. Conversely, in the case of using the I-80 data, the model exhibits its best predictive performance with α=1/2 and β=3/2, correctly forecasting 171 instances of “Merging” actions and 268 instances of “Waiting” actions. These results indicate the model’s effectiveness in predicting mandatory lane-changing interactions at freeway on-ramps and its robust predictive capabilities for each strategy profile.

**Fig 7:**
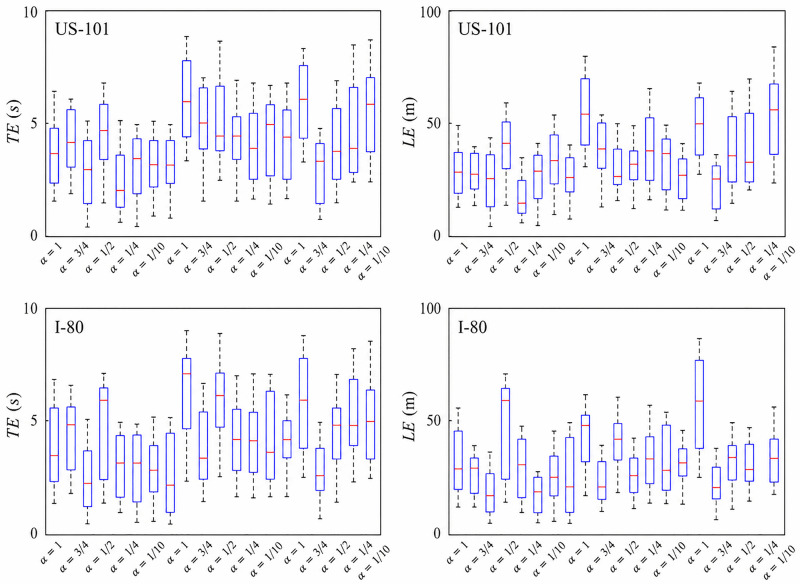
Lane-changing time and location error.

To further evaluate the model’s performance across different datasets and data qualities, this study analyses time and location errors, as depicted in Fig 7. The average TE and LE for US-101 across various scenarios range from 1.71s to 5.99s and from 10.38m to 51.97m, respectively. Similarly, for I-80, the average TE and LE across different cases vary from 1.85s to 7.01s and from 12.67m to 54.73m, respectively. These findings highlight the model’s robust predictive capabilities and its effectiveness in capturing merging behaviour at freeway on-ramps across diverse datasets and data qualities.

### 5.3 Comparison of different models

In this section, this study compares the proposed model with three other mandatory lane-changing models based on game theory that utilize the Nash Equilibrium (NE) solution. These models, proposed by Kita (1999) [13], Liu et al. (2007) [17], and Ali et al. (2019) [35], are denoted as Kita’s model, Liu’s model, and AZHW model. To assess the performance of these models, this study utilizes metrics including MAE, RMSE, DR, FAR, TE, and LE using US-101 and I-80 data. The results are summarized in Tables 6 and 7.

**Table 6:** Comparison results using US-101 data.

	Our model	Kita’s model	Liu’s model	AZHW model
MAE	0.11	0.23	0.22	0.19
RMSE	0.31	0.48	0.47	0.43
DR (%)	91.47	77.24	79.27	82.49
FAR (%)	8.53	22.76	20.73	17.51
TE (s)	1.71	6.19	6.06	3.68
LE (m)	10.38	39.17	37.87	24.94

**Table 7:** Comparison results using I-80 data.

	Our model	Kita’s model	Liu’s model	AZHW model
MAE	0.10	0.21	0.19	0.16
RMSE	0.32	0.44	0.42	0.37
DR (%)	92.04	76.97	79.23	84.74
FAR (%)	7.96	23.03	20.77	15.26
TE (s)	1.85	5.36	6.53	2.17
LE (m)	12.67	36.72	43.87	14.29

Table 6 presents the MAE values, which are 0.11 for our model, 0.23 for Kita’s model, 0.22 for Liu’s model, and 0.19 for the AZHW model, respectively. The corresponding RMSE values are 0.31, 0.48, 0.47, and 0.43, respectively. Our model achieves a DR of 91.47%, while Kita’s model, Liu’s model, and the AZHW model have DR values of 77.24%, 79.27%, and 82.49%, respectively. Additionally, our model exhibits lower TE, and LE compared to the other three models, indicating its superior accuracy in predicting mandatory lane-changing behaviour.

Similarly, when using the I-80 data, our model outperforms the other three models in all aspects, as shown in Table 7. The validation results indicate that our model excels compared to the other three models, demonstrating superior predictive capabilities and more accurate predictions of lane-changing behaviour. These outcomes can be attributed to the absence of the adaptive value function and probability weighting function in the payoff functions of the other three models, leading to increased prediction errors.

Moreover, our model’s enhanced performance can be attributed to its comprehensive consideration of variables, including the incorporation of loss aversion and recognition of varying risk attitudes towards gains and losses. By integrating this aspect, our model more effectively captures drivers’ decision-making processes, resulting in more precise and reliable predictions of lane-changing behaviour. In summary, the validation results emphasize the superiority of our model compared to Kita’s model, Liu’s model, and the AZHW model. This highlights its potential as a more efficient and realistic approach for researching and understanding mandatory lane-changing behaviour at freeway on-ramps.

## 6. Conclusions

This study introduces a novel mandatory lane-changing model grounded in prospect theory, utilizing a game-theoretical approach. Within this framework, drivers’ responses are influenced more by perceived safety losses than equivalent efficiency gains. The model incorporates payoff functions for both the on-ramp vehicle and the mainline vehicle, considering both efficiency gains and safety losses. It employs a non-linear probability weighting scheme rooted in loss aversion. To estimate model parameters, vehicle trajectory data from NGSIM is utilized, leveraging a bilevel optimization approach. In this approach, the upper level minimizes the squared difference between observed and predicted actions through a non-linear programming problem, while the lower level seeks the Nash equilibrium solution.

Validation results demonstrate the model’s close alignment with empirical data, highlighting its robust predictive capacity in describing mandatory lane-changing behaviour. Compared to three other game theory-based models, our model exhibits lower values for MAE, RMSE, FAR, TE, and LE, indicating superior predictive performance. These findings indicate improved predictive performance of our model in accurately characterizing drivers’ mandatory lane-changing behaviour. In conclusion, the utilization of our model enriches our understanding of driver decision-making dynamics during lane-changing manoeuvres and contributes to the development of more effective traffic safety and management strategies.

Recent data-driven and deep-learning approaches provide strong prediction performance by learning nonlinear lane-changing patterns from large trajectory datasets. Nevertheless, the proposed model differs from these methods by explicitly representing the strategic interaction between conflicting drivers and by providing interpretable behavioural parameters related to efficiency gain, safety loss, loss aversion, and probability weighting. This distinction is particularly useful when the modelling objective is not only prediction but also behavioural interpretation and policy-oriented safety analysis.

Several limitations should be acknowledged. First, the empirical analysis is based on two historical NGSIM freeway merging sites, and the transferability of the calibrated parameters to other roadway geometries, traffic compositions, and connected/automated driving environments should be further examined. Second, the present formulation uses a two-player complete-information game between OV and MV, whereas real merging behaviour may involve multiple surrounding vehicles and imperfect perception. Third, the strategy space is discretised into merging/ waiting and accelerating/ maintaining/ decelerating, which improves interpretability but cannot fully represent continuous control decisions. Fourth, safety loss is represented mainly by TTC-based indicators; future work may incorporate additional conflict indicators, such as post-encroachment time, deceleration rate to avoid collision, and surrogate safety measures based on predicted trajectories. Finally, the model infers risk-related behavioural parameters from trajectories rather than from direct driver demographic or psychological observations. Future research could extend the framework to multi-agent incomplete-information games and hybrid interpretable-data-driven models.

## References

[1] Patire AD, Cassidy MJ. Lane changing patterns of bane and benefit: observations of an uphill expressway. Transp Res B: Methodol. 2011;45(4):656–66. doi: 10.1016/j.trb.2011.01.003

[2] Cassidy MJ, Rudjanakanoknad J. Increasing the capacity of an isolated merge by metering its on-ramp. Transp Res B: Methodol. 2005;39(10):896–913. doi: 10.1016/j.trb.2004.12.001

[3] Laval JA, Daganzo CF. Lane-changing in traffic streams. Transp Res B: Methodol. 2006;40(3):251–64. doi: 10.1016/j.trb.2005.04.003

[4] Ji A, Levinson D. A review of game theory models of lane changing. Transp A: Transp Sci. 2020;16(3):1628–47.

[5] Gao J, Yi J, Murphey YL. Joint learning of video images and physiological signals for lane-changing behavior prediction. Transp A: Transp Sci. 2022;18(3):1234–53.

[6] Ali Y, Zheng Z, Haque MdM. Connectivity’s impact on mandatory lane-changing behaviour: evidences from a driving simulator study. Transp Res C: Emerg Technol. 2018;93:292–309. doi: 10.1016/j.trc.2018.06.008

[7] Gipps PG. A model for the structure of lane-changing decisions. Transp Res B: Methodol. 1986;20(5):403–14. doi: 10.1016/0191-2615(86)90012-3

[8] Hidas P. Modelling lane changing and merging in microscopic traffic simulation. Transp Res C: Emerg Technol. 2002;10(5–6):351–71. doi: 10.1016/s0968-090x(02)00026-8

[9] Kesting A, Treiber M, Helbing D. General lane-changing model MOBIL for car-following models. Transp Res Rec. 2007;1999(1):86–94. doi: 10.3141/1999-10

[10] Ahmed KI. Modeling drivers’ acceleration and lane changing behavior [Doctoral dissertation]. Massachusetts Institute of Technology; 1999.

[11] Toledo T, Koutsopoulos HN, Ben-Akiva ME. Modeling integrated lane-changing behavior. Transp Res Rec. 2003;1857(1):30–8. doi: 10.3141/1857-04

[12] Xu T, Zhang Z, Wu X, Qi L, Han Y. Recognition of lane-changing behaviour with machine learning methods at freeway off-ramps. Phys A: Stat Mech Appl. 2021;567:125691. doi: 10.1016/j.physa.2020.125691

[13] Kita H. A merging–giveway interaction model of cars in a merging section: a game theoretic analysis. Transp Res A: Policy Pract. 1999;33(3–4):305–12. doi: 10.1016/s0965-8564(98)00039-1

[14] Kita H, Tanimoto K, Fukuyama K. A game theoretic analysis of merging-giveway interaction: a joint estimation model. Transportation and Traffic Theory in the 21st Century. Emerald Group Publishing Limited; 2002. p. 503–18.

[15] Talebpour A, Mahmassani HS, Hamdar SH. Modeling lane-changing behavior in a connected environment: a game theory approach. Transp Res Procedia. 2015;7:420–40. doi: 10.1016/j.trpro.2015.06.022

[16] Nash J. Non-cooperative games. Ann Math. 1951;54(2):286. doi: 10.2307/1969529

[17] Liu HX, Xin W, Adam Z, Ban J. A game theoretical approach for modelling merging and yielding behaviour at freeway on-ramp sections. International Symposium on Transportation and Traffic Theory. 2007;3:197–211.

[18] Kang K, Rakha HA. Game theoretical approach to model decision making for merging maneuvers at freeway on-ramps. Transp Res Rec. 2017;2623(1):19–28. doi: 10.3141/2623-03

[19] Kahneman D, Tversky A. Prospect theory: an analysis of decision under risk. Econometrica. 1979;47(2):263. doi: 10.2307/1914185

[20] Yang Q, Koutsopoulos HN. A Microscopic traffic simulator for evaluation of dynamic traffic management systems. Transp Res C: Emerg Technol. 1996;4(3):113–29. doi: 10.1016/s0968-090x(96)00006-x

[21] Hidas P. Modelling vehicle interactions in microscopic simulation of merging and weaving. Transp Res C: Emerg Technol. 2005;13(1):37–62. doi: 10.1016/j.trc.2004.12.003

[22] Ahmed K, Ben-Akiva M, Koutsopoulos H, Mishalani R. Models of freeway lane changing and gap acceptance behavior. Transp Traffic Theory. 1996:13:501–15.

[23] Toledo T, Katz R. State dependence in lane-changing models. Transp Res Rec. 2009;2124(1):81–8. doi: 10.3141/2124-08

[24] Jin C-J, Knoop VL, Li D, Meng L-Y, Wang H. Discretionary lane-changing behavior: empirical validation for one realistic rule-based model. Transp A: Transp Sci. 2018;15(2):244–62. doi: 10.1080/23249935.2018.1464526

[25] Li K, Wang X, Xu Y, Wang J. Lane changing intention recognition based on speech recognition models. Transp Res C: Emerg Technol. 2016;69:497–514. doi: 10.1016/j.trc.2015.11.007

[26] Suh J, Chae H, Yi K. Stochastic model-predictive control for lane change decision of automated driving vehicles. IEEE Trans Veh Technol. 2018;67(6):4771–82. doi: 10.1109/tvt.2018.2804891

[27] Alexiadis V, Colyar J, Halkias J, Hranac R, McHale G. The next generation simulation program. ITE J. 2004;74(8):22.

[28] Hamdar SH. Modeling driver behavior as a stochastic hazard-based risk-taking process. Civ Environ Eng. 2009:158–68.

[29] Balal E, Cheu RL, Sarkodie-Gyan T. A binary decision model for discretionary lane changing move based on fuzzy inference system. Transp Res C: Emerg Technol. 2016;67:47–61. doi: 10.1016/j.trc.2016.02.009

[30] Hou K, Zheng F, Liu X, Fan Z. Cooperative vehicle platoon control considering longitudinal and lane-changing dynamics. Transp A: Transp Sci. 2023:1–29.

[31] Ahmad F, Almarri O, Shah Z, Al-Fagih L. Game theory applications in traffic management: a review of authority-based travel modelling. Travel Behav Soc. 2023;32:100585. doi: 10.1016/j.tbs.2023.100585

[32] Wu Y, Li H. Signalling security: an observational and game theory approach to inter-pedestrian psychology. Transp Res F: Traffic Psychol Behav. 2022;86:238–51.

[33] Wang M, Hoogendoorn SP, Daamen W, van Arem B, Happee R. Game theoretic approach for predictive lane-changing and car-following control. Transp Res C: Emerg Technol. 2015;58:73–92. doi: 10.1016/j.trc.2015.07.009

[34] Yao R, Du X. Modelling lane changing behaviors for bus exiting at bus bay stops considering driving styles: a game theoretical approach. Travel Behav Soc. 2022;29:319–29. doi: 10.1016/j.tbs.2022.07.008

[35] Arbis D, Dixit VV. Game theoretic model for lane changing: incorporating conflict risks. Accid Anal Prev. 2019;125:158–64. doi: 10.1016/j.aap.2019.02.007 30763813

[36] Ji A, Levinson D. Estimating the social gap with a game theory model of lane changing. IEEE Trans Intell Transport Syst. 2021;22(10):6320–9. doi: 10.1109/tits.2020.2991242

[37] Sheikh MS, Wang J, Regan A. A game theory-based controller approach for identifying incidents caused by aberrant lane changing behavior. Phys A: Stat Mech Appl. 2021;580:126162. doi: 10.1016/j.physa.2021.126162

[38] Wang B, Li Z, Wang S, Li M, Ji A. Modeling bounded rationality in discretionary lane change with the quantal response equilibrium of game theory. Transp Res B: Methodol. 2022;164:145–61. doi: 10.1016/j.trb.2022.08.008

[39] Shimojo A, Ninomiya Y, Miwa K, Terai H, Matsubayashi S, Okuda H, et al. How impressions of other drivers affect one’s behavior when merging lanes. Transp Res F: Traffic Psychol Behav. 2022;89:236–48.

[40] Venthuruthiyil SP, Chunchu M. Interrupted and uninterrupted lane changes: a microscopic outlook of lane-changing dynamics. Transp A: Transp Sci. 2022;18(3):1679–98.

[41] Ali Y, Zheng Z, Haque MdM, Wang M. A game theory-based approach for modelling mandatory lane-changing behaviour in a connected environment. Transp Res C: Emerg Technol. 2019;106:220–42. doi: 10.1016/j.trc.2019.07.011

[42] Ozaki H. Reaction and anticipation in the car following behavior. Proceedings of the 13th International Symposium on Traffic and Transportation Theory; 1993.

[43] Ali Y, Zheng Z, Haque MdM, Yildirimoglu M, Washington S. CLACD: a complete LAne-Changing decision modeling framework for the connected and traditional environments. Transp Res C: Emerg Technol. 2021;128:103162. doi: 10.1016/j.trc.2021.103162

[44] Ali Y, Zheng Z, Bliemer MCJ. Calibrating lane-changing models: two data-related issues and a general method to extract appropriate data. Transp Res C: Emerg Technol. 2023;152:104182. doi: 10.1016/j.trc.2023.104182

[45] Lin D, Li L, Jabari SE. Pay to change lanes: a cooperative lane-changing strategy for connected/automated driving. Transp Res C: Emerg Technol. 2019;105:550–64. doi: 10.1016/j.trc.2019.06.006

[46] Hamdar SH, Treiber M, Mahmassani HS, Kesting A. Modeling driver behavior as sequential risk-taking task. Transp Res Rec. 2008;2088(1):208–17.

[47] Talebpour A, Mahmassani HS, Hamdar SH. Multiregime sequential risk-taking model of car-following behavior: specification, calibration, and sensitivity analysis. Transp Res Rec. 2011;2260(1):60–6.

[48] Tversky A, Kahneman D. Advances in prospect theory: cumulative representation of uncertainty. J Risk Uncertain. 1992;5(4):297–323. doi: 10.1007/bf00122574

[49] Camerer C, Lovallo D. Overconfidence and excess entry: an experimental approach. Am Econ Rev. 1999;89(1):306–18. doi: 10.1257/aer.89.1.306

[50] Sharot T, Riccardi AM, Raio CM, Phelps EA. Neural mechanisms mediating optimism bias. Nature. 2007;450(7166):102–5. doi: 10.1038/nature06280 17960136

[51] Yao J, Li D. Bounded rationality as a source of loss aversion and optimism: a study of psychological adaptation under incomplete information. J Econ Dyn Control. 2013;37(1):18–31. doi: 10.1016/j.jedc.2012.07.002

[52] Herold F, Netzer N. Second-best probability weighting. Games Econ Behav. 2023;138:112–25. doi: 10.1016/j.geb.2022.12.005

[53] Montanino M, Punzo V. Trajectory data reconstruction and simulation-based validation against macroscopic traffic patterns. Transp Res B: Methodol. 2015;80:82–106. doi: 10.1016/j.trb.2015.06.010

[54] Zheng Z. Recent developments and research needs in modeling lane changing. Transp Res B: Methodol. 2014;60:16–32. doi: 10.1016/j.trb.2013.11.009

